# Relief as a Reward: Hedonic and Neural Responses to Safety from Pain

**DOI:** 10.1371/journal.pone.0017870

**Published:** 2011-04-07

**Authors:** Siri Leknes, Michael Lee, Chantal Berna, Jesper Andersson, Irene Tracey

**Affiliations:** Nuffield Division of Anaesthetics, Nuffield Department of Clinical Neurosciences, Centre for Functional Magnetic Resonance Imaging of the Brain (FMRIB), University of Oxford, Oxford, Oxfordshire, United Kingdom; Pontifical Catholic University of Rio Grande, Brazil

## Abstract

Relief fits the definition of a reward. Unlike other reward types the pleasantness of relief depends on the violation of a negative expectation, yet this has not been investigated using neuroimaging approaches. We hypothesized that the degree of negative expectation depends on *state* (dread) and *trait* (pessimism) sensitivity. Of the brain regions that are involved in mediating pleasure, the nucleus accumbens also signals unexpected reward and positive prediction error. We hypothesized that accumbens activity reflects the level of negative expectation and subsequent pleasant relief. Using fMRI and two purpose-made tasks, we compared hedonic and BOLD responses to relief with responses during an appetitive reward task in 18 healthy volunteers. We expected some similarities in task responses, reflecting common neural substrates implicated across reward types. However, we also hypothesized that relief responses would differ from appetitive rewards in the nucleus accumbens, since only relief pleasantness depends on negative expectations. The results confirmed these hypotheses. Relief and appetitive reward task activity converged in the ventromedial prefrontal cortex, which also correlated with appetitive reward pleasantness ratings. In contrast, dread and pessimism scores correlated with relief but not with appetitive reward hedonics. Moreover, only relief pleasantness covaried with accumbens activation. Importantly, the accumbens signal appeared to specifically reflect individual differences in anticipation of the adverse event (dread, pessimism) but was uncorrelated to appetitive reward hedonics. In conclusion, relief differs from appetitive rewards due to its reliance on negative expectations, the violation of which is reflected in relief-related accumbens activation.

## Introduction

Relief from pain fits with the definition of a reward [1], [2]. Like other reward types, relief (reward induced though omission or reduction of an aversive event) can be pleasurable [3]. Similarities in behavioural and neural responses to relief and appetitive rewards have been shown across species and tasks [1], [4], [5], [6], [7]. For instance, monetary gain and omission of monetary loss activated overlapping regions in the ventromedial prefrontal cortex [8], [9]. Interestingly, appetitive rewards are more enjoyable when also providing relief (e.g. when food relieves hunger) [10]. Therefore, it appears that the hedonic aspects of appetitive reward and relief can be additive.

However, an important difference exists between relief and other reward types. Unlike appetitive rewards, pleasure from relief is derived from a violation of a negative expectation. Whereas the pleasure of an appetitive reward depends on characteristics of the rewarding stimulus itself, relief pleasantness correlates with the aversiveness of pain [3]. Of the brain regions that are involved in mediating pleasure, the nucleus accumbens (NAc) also serves to signal unexpected reward and positive prediction error [7], [11], [12], [13]. We therefore hypothesized that activity in this region would reflect the pleasure of relief, but not the pleasure of appetitive rewards. Furthermore, we predicted that only during relief would activity in the NAc depend on state and trait influences on the expectation of relief. Specifically, NAc activity would differ between optimists and pessimists, because pessimists would dread the anticipated adversity more, and not expect to escape adversity.

We designed two functional MRI tasks to elucidate similarities and differences between hedonic and neural responses during relief and appetitive rewards.

**Figure 1:**
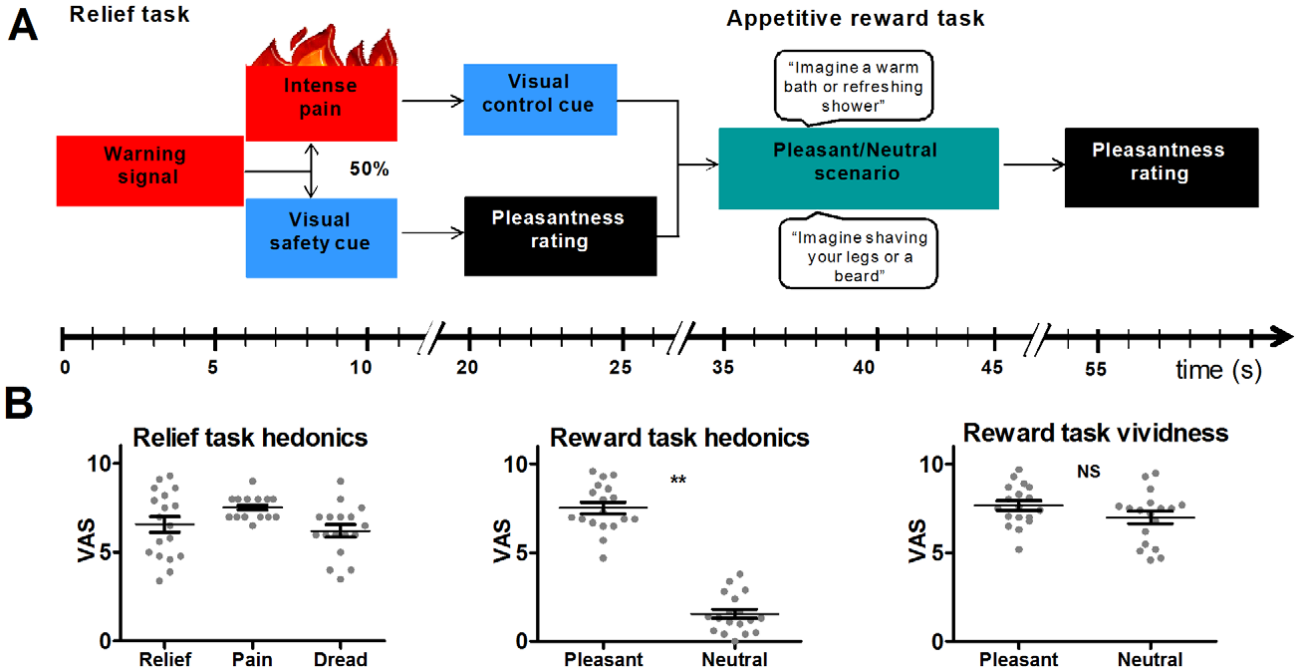
Experimental design and behavioural ratings. A: Overview of relief and appetitive reward task design and timing of events. Each relief task trial began with a 6-second anticipatory period, in which participants expected intense pain. 50% of anticipation periods were followed by intense pain for 5 seconds; in the remaining events a safety cue was presented on the screen for 5 seconds. In the appetitive reward task, participants imagined pleasant scenarios (activities involving appetitive rewards) or neutral scenarios according to the text on the screen. The graphs in panel B depict behavioural ratings from the relief and the appetitive reward tasks. ** denotes p<0.001. Error bars indicate SEM.

In the relief task, a warning signal was 50% predictive of intense pain (Figure 1A). After the warning, participants experienced either pain or a safety cue, which signalled that the impending pain had been omitted. Participants rated pleasantness of relief following the safety cue; they also reported their level of anticipatory dread. In the appetitive reward task, participants imagined scenarios where only the hedonic value (pleasant or neutral) varied between the main task and the control condition. This design was based on previous studies showing that imagination alone is sufficient to activate brain regions associated with perception or execution of various sensory/motor events [14], [15], [16].

We expected relief to share hedonic and neural features with appetitive rewards. However, we hypothesised that unlike appetitive rewards, relief would also be influenced by the degree of negative expectation, which varies according to state (dread) and trait sensitivity to negative expectation (pessimism).

## Methods

### Participants

Eighteen healthy, right-handed participants (mean age 28 years, range 20 to 36 years, 9 females) were recruited for this study. All participants gave written informed consent, and underwent comprehensive screening to ensure the absence of contraindications to MRI. Participants were reimbursed 25 GBP for their time and travel expenses. The study was approved by the Central Oxfordshire Clinical Research Ethics Committee (C02.286; Mapping brain function with functional magnetic resonance imaging) and conformed to the guidelines of the Declaration of Helsinki (1996).

### Experimental design

The study design is outlined in Figure 1A. The relief task and the appetitive reward task each consisted of 20 trials which were presented in pseudorandomised order with no more than two subsequent repeats of the same condition within each task. Trials of the two tasks were intermixed during the scan, such that each relief trial was followed by an appetitive reward task trial, which was followed by another relief task trial, and so on. The total duration of the fMRI experiment was 25 min.

#### Relief task

Each trial in the relief task started with a 6-second pain cue, paired with a subsequent 5-second painful heat stimulus (individually calibrated to induce intense pain) or a visual safety cue displayed for 5 seconds (figure 1A). In 50% of trials, the warning cue was followed by a painful heat stimulus, and in the remaining 10 trials it was followed by a safety cue. This partial trial design ensures that signal changes elicited by the warning signal can be disentangled from activity changes caused by subsequent heat pain or relief. Nine seconds after the safety cue, participants rated relief pleasantness on a visual analogue scale displayed for 6 seconds (“How pleasant was the relief you felt?/neutral/intensely pleasant”). Nine seconds after the painful heat stimulus, when no further pain was expected, a control cue was displayed for 5 seconds. In the first two relief task trials, the warning signal was always followed by the painful heat stimulus; this was done to ensure that each safety cue would elicit relief.

We used an in-house thermal resistor [3], [17], [18], [19] to deliver noxious thermal stimulation (5 seconds at the designated temperature) to the volar aspect of the participant's left arm. Stimulus temperatures were determined on an individual basis after the participants had been placed in the scanner, but before the onset of the experiment. With each stimulus presentation the temperature was increased, until participants reported a pain intensity of 8 on a standard 11-point numerical rating scale (NRS) where 0 is no pain, 1 is the pain threshold and 10 is extreme pain. The interstimulus interval was at least 60 seconds during temperature calibration and during the experiment to avoid skin damage and sensitisation. The average temperature required to cause intense pain sensation was 49.2±1.9°C (mean ± SD). A total of 10 noxious heat stimuli were delivered during the experiment.

The visual cues in the relief task consisted of coloured rectangles with the following text: ‘Heat stimulus coming up’ (warning signal, 6 s); ‘No heat stimulus’ (safety cue, 5 s) and ‘No stimulus’ (control cue, 5 s). The pain cue was always displayed in red. The blue and violet display colours of the safety and control cues were counterbalanced across participants.

Ratings of pain intensity of pain and dread were collected during debriefing using the standard 11-point numerical rating scale (NRS) with anchors “no pain/intense pain”, “no dread/intense dread”.

#### Appetitive reward task

In the appetitive reward task trials, written descriptions of scenarios, 10 pleasant and 10 neutral, were displayed for 10 seconds each (figure 1A). Participants were instructed to imagine each scenario for as long as the words remained on the screen. The pleasant scenarios described activities involving appetitive rewards. The pleasant and neutral imagined scenarios differed only in the pleasantness elicited during the task. Therefore, contrasting activity during pleasant and neutral scenarios yielded an activation map corresponding to appetitive reward-induced activation.

Nine seconds after each scenario, participants rated imagined pleasantness on a visual analogue scale displayed for 6 seconds (“How pleasant was the scenario you imagined?/neutral/intensely pleasant”). This rating scale was followed by another 9-second delay before another trial commenced. The scenarios were displayed on the screen with a coloured rectangle framing the text, indicating whether the scenario was pleasant or neutral. The rectangles were either dark blue or green in colour; this was counterbalanced across participants.

The pleasant scenarios were adapted from the Snaith-Hamilton Pleasure Scale (SHAPS) [20]. The advantage of using these scenarios in an appetitive reward task is twofold: 1) The scenarios were specifically designed to assess hedonic capacity; and 2) The scenarios describe a range of sensory and information-induced rewards that are commonly experienced. Therefore, the brain regions associated with activation across this range of rewarding scenarios should yield a purer estimate of positive hedonic experience than a contrast based upon only one type of stimulus, such as a monetary reward task.

A pool of 34 neutral sentences designed to closely match the 14 pleasant questionnaire items in content and word length was generated. In a pilot experiment, 14 participants (mean age 35, age range 25–45, 8 males) were asked to imagine each scenario in turn, and to rate pleasantness/unpleasantness on an 11-point numerical rating scale, where -5 is most unpleasant, 0 is neutral, and +5 is most pleasant). Participants also reported how well they were able to imagine that particular scenario (vividness) on a 6-point numerical rating scale where 0 is not at all, and 5 is very well. On the basis of the pilot data, 10 pleasant and 10 neutral sentences were selected for use in the experiment (Table 1). The two condition scenarios were matched for vividness and number of words; consideration was given to matching sentence complexity, the predominant imagined sensory modality, and the ‘everyday’ nature of the SHAPS items, although no specific ratings were acquired for these factors.

**Table 1:** Pleasant and neutral scenarios used in the experiment.

Imagined scenarios	Pleasantness ratings (−5 to 5)	Vividness ratings (0–5)	Number of words in sentence
**Neutral scenarios**			
Imagine brushing your teeth	**0.9**	**4.3**	4
Imagine shaving (your legs or a beard)	**−0.4**	**4.2**	7
Imagine washing up a mug	**−0.1**	**4.1**	5
Imagine peeling an orange	**0.4**	**3.9**	4
Imagine having a meal on an airplane	**0.4**	**3.9**	6
Imagine looking at other people on a train or a bus	**0.4**	**3.8**	11
Imagine being given your change at the supermarket	**0.1**	**3.9**	8
Imagine the sound of walking on gravel	**0.9**	**3.9**	7
Imagine waiting for the kettle to boil	**−0.8**	**4.0**	7
Imagine drinking lukewarm water	**−0.4**	**3.5**	4
Mean	**0.1**	**4.0**	6.3
Standard deviation	0.6	0.2	2.2
**Pleasant scenarios**			
Imagine a warm bath or refreshing shower	**3.8**	**4.3**	7
Imagine a bright sunny day	**3.9**	**4.3**	5
Imagine doing one of your hobbies and pastimes	**4.4**	**4.5**	8
Imagine the smell of a fresh sea breeze or freshly baked bread	**3.9**	**4.3**	12
Imagine having your favourite meal	**4.5**	**4.7**	5
Imagine seeing other people's smiling faces	**3.0**	**3.7**	6
Imagine helping others	**3.6**	**3.7**	3
Imagine watching your favourite TV programme	**3.5**	**3.8**	6
Imagine a beautiful landscape or view	**3.4**	**3.9**	6
Imagine drinking a cup of tea or coffee or your favourite drink	**3.6**	**4.5**	12
Mean	**3.8**	**4.2**	7
Standard deviation	0.5	0.4	2.9

The pleasant scenarios were adapted from the Snaith-Hamilton Pleasure scale. On the basis of the pilot data, the 11 pleasant and 11 neutral sentences displayed above were selected for use in the experiment. The two condition scenarios were matched for vividness and number of words.

During pre-scan testing immediately before the fMRI session, participants rated the vividness of each imagined scenario on a visual analogue scale (“How well did you imagine the scenario?/not at all/intensely”). The pre-scan testing consisted of a 10-minute session outside the scanner, and was conducted in order to familiarise the participants with the task of imagining the pleasant and neutral scenarios. This session also enabled us to obtain vividness ratings from each scenario from the same participants without adding extra time and complexity to the tasks performed in the fMRI environment. The pleasant and neutral scenarios were displayed on a computer screen for 10 seconds each in pseudorandomised order as described above. After a 4-second interval, a VAS was displayed on the screen for 6 seconds (“How well were you able to imagine the scenario/not at all/intensely”). The two first scenarios (one pleasant, one neutral) were included for practice only and were not used in the fMRI part of the experiment; the remaining 20 scenarios were used both during pre-scan testing and during the appetitive reward task in the scanner.

### State and trait measures

To assess trait optimism/pessimism, participants were asked to complete the Life Orientation Test-Revised (LOT-R) [21], which measures disposition on a single scale.

### MRI data acquisition

Functional imaging data was acquired in a 3 Tesla human Varian MRI system (Oxford Magnet Technology, 1 m bore, Oxford, UK) using a bird-cage radio frequency coil for pulse transmission and signal detection by a reduced bore gradient coil (Magnex SGRAD MK III, Oxford, UK). A gradient echo-planar imaging (EPI) sequence with TR = 3 s; matrix = 64×64; TE = 30 ms; 41 axial oblique slices; volumes = 494 (the first four were ‘dummy’ scans); FOV = 192×192; voxel size = 3 mm^3^ was used. Functional scans were acquired continuously throughout each scan. In addition, a T1-weighted structural scan (voxel size 1 mm^3^) was acquired to improve registration to standard space.

### Behavioural data analysis

Statistical analysis of behavioural and questionnaire data was performed using SPSS 12.0 (SPSS Inc., Chicago). Pearson's correlation test was used to test for significant correlations between the (normally distributed) behavioural measures. For the LOT-R, the total score for optimism- and pessimism-related items was used [22]. Paired t-tests were used to assess differences between pleasant and neutral scenarios in the appetitive reward task. Differences between more and less pessimistic participants in our sample, as defined by a median split, were assessed using two-sample t-tests.

### fMRI data analysis

fMRI data analysis was performed in a multi-stage process using FEAT [functional MRI (fMRI) Expert Analysis Tool] Version 5.92, part of FSL [Oxford Centre for Functional Magnetic Resonance Imaging of the Brain (FMRIB, Oxford, UK) Software Library; www.fmrib.ox.ac.uk/fsl]. Pre-statistics processing was applied as follows; motion correction using MCFLIRT[23]; non-brain removal using BET (Brain Extraction Tool) [24]; spatial smoothing using a Gaussian kernel of full-width-half-maximum 5 mm; highpass temporal filtering (Gaussian-weighted least-squares straight line fitting, with sigma = 50.0 s). Input stimulus functions were defined for each visual cue type (pain, safety and control), for the painful heat stimulation, for the pleasant and neutral written scenarios as well as for the tasks of rating. Input stimulus functions were convolved with the gamma HRF (mean lag 6 s and full-width-at-half-height 6 s) to yield regressors for the general linear model (GLM). The estimated motion parameters for each participant were included as covariates of no interest to reduce spurious activations due to head motion and scanner drift, thereby increasing statistical sensitivity. Time-series statistical analysis was carried out using FILM with local autocorrelation correction [25]. Registration to high resolution structural and standard MNI space images was performed using FLIRT [23], [26].

Higher-level (group) statistical analysis was performed using FLAME (FMRIB's Local Analysis of Mixed Effects) [27], [28], which produced Z (Gaussianised T/F) statistic images. Only voxels of Z>2.3 were further submitted to cluster-based correction (p<0.05) [29] for multiple voxel-wise comparisons (correcting for multiple comparisons). Small volume corrections employing a voxel-based approach (p<0.05) were used for additional region of interest (ROI) analyses [30]. ROIs of the left and right nucleus accumbens were generated in standard space using the Harvard-Oxford Subcortical Structural Probabilistic Atlas (FMRIB, Oxford, UK; and Harvard Center for Morphometric Analysis, Charlestown MA, USA).

**Figure 2:**
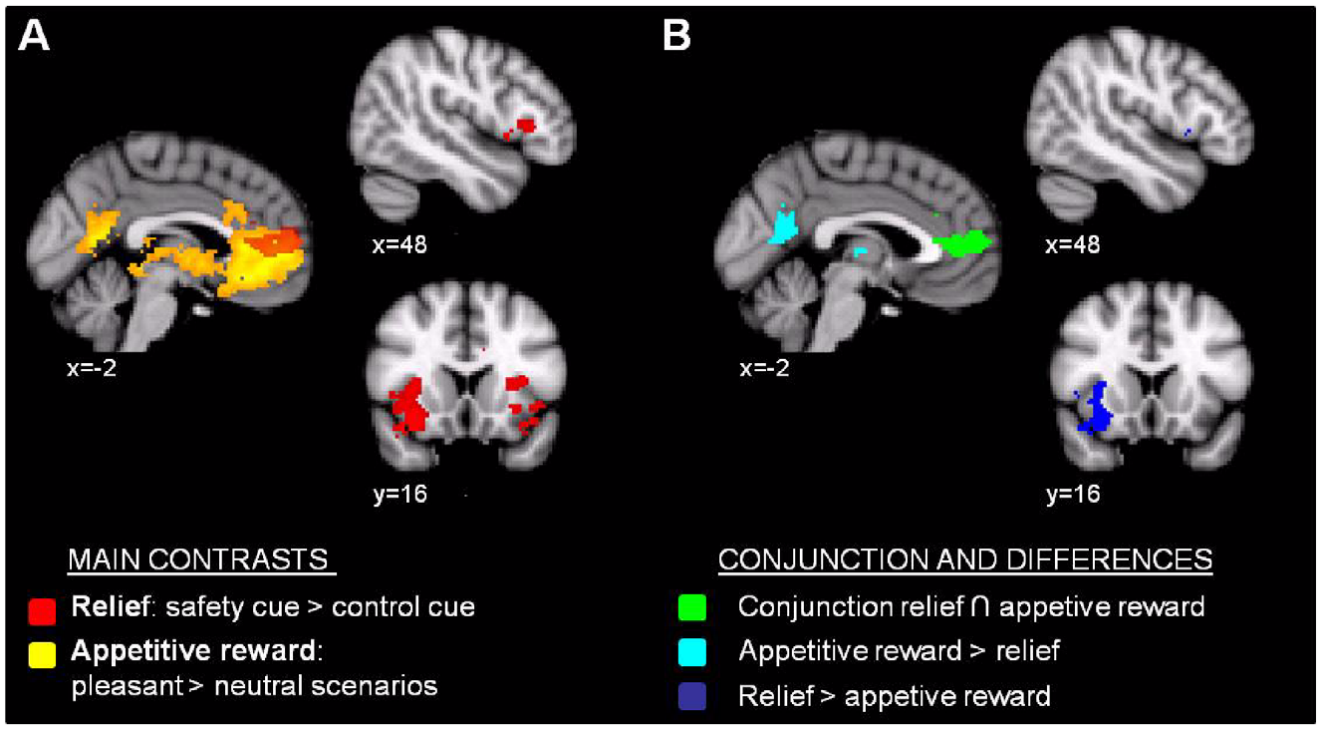
Task-induced brain activation patterns. A: Main effect of relief task (red) and appetitive reward task (yellow). B: The overlap between the two tasks (green) and between-tasks contrasts (light and dark blue). MRI images are overlaid on the normalised average of the whole group's structural scans, and thresholded using a cluster-based approach with Z>2.3, p<0.05, corrected for multiple comparisons.

At the group level the following analyses were performed:

1. Main effect of the relief task and main effect of the appetitive reward task.The main effect of relief within a given participant was assessed by the contrast [safety cue – control cue]. This was averaged across all participants (optimists and pessimists) at the group level. Similarly, the effect of appetitive reward was assessed for each participant using the contrast [imagined pleasant scenarios – imagined neutral scenarios] and averaged across all participants at the group level. Since there was a (nonsignificant) trend towards higher vividness ratings for the pleasant compared to the neutral scenarios, which represented a potential confound for the appetitive reward contrast, we included as a covariate of no interest a regressor based on each participant's difference in reported vividness for the two scenario types. The resulting maps (with voxels that survived threshold for inference) are shown in Figure 2.
2. Comparison of the relief task and appetitive reward task activation patterns.To identify areas where significant activity was elicited by both tasks, a conjunction analysis was performed using the main effect thresholded statistical maps from the relief and reward tasks identified in the above analysis (these were binarised and multiplied with each other). To identify areas where activity in the two tasks differed significantly, we performed a paired t-test comparing the main effect of the relief task and the reward task for each participant. Since we were interested in identifying differences between relief and reward activity in the regions identified as showing a main effect of either relief or reward, the results from the relief>reward analysis were masked by the thresholded statistical map showing significant positive relief activation. Similarly, the thresholded statistical map from the reward>relief contrast was masked by the main effect of the appetitive reward map, so that only regions which showed significant positive activation during the reward task *and* significantly higher activity in the reward task relative to the relief task, remained.
3. Activation covarying with behavioural ratingsThis set of analysis steps used each participant's average rating from the relief and appetitive reward tasks as regressors in the GLM analysis. For the relief task, we investigated correlations between activation in the contrast safety cue>control cue and each participant's score on relief pleasantness, dread and optimism/pessimism, whilst modelling out the main effect of group. For the appetitive reward task, we tested the correlation of pleasantness ratings with the main contrast (pleasant>neutral scenarios). A priori regions of interest (left and right NAc and the ventromedial prefrontal area activated by both tasks) were used for small volume correction using voxel-wise thresholding at p<0.05, corrected for multiple comparisons.
4. Creating peri-stimulus plots to illustrate NAc responsesSemi-spherical ROI masks used in this analysis were generated for each individual's left and right NAc by linear transformation of the voxels with MNI coordinates 8, 10, −10 (right) and −8, 10, −10 (left) into individual EPI space [23]. The resulting masks were visually inspected to ensure that composite voxels were in the correct anatomical regions. The mean timeseries of the left and right NAc masks were extracted for each participant using Featquery [www.fmrib.ox.ac.uk/featquery].

## Results

### Behavioural results

As illustrated in Figure 1B, and demonstrated by subjective reports of relief pleasantness (6.6±1.9), pain intensity (7.5±0.6), and anticipatory dread (6.3±1.4, mean±SD), the relief paradigm successfully elicited the expected positive and negative hedonic feelings during the experiment. Similarly, in the appetitive reward task the scenarios adapted from the Snaith-Hamilton pleasure scale elicited a high pleasantness score (7.5±1.3), while the neutral control scenarios raised a significantly lower pleasantness rating (1.6±1.1, p<0.001); the difference between the pleasantness ratings from the pleasant and neutral scenarios (6.0±1.7, mean±SD) in the appetitive reward task was used in subsequent analyses. Participants reported little difficulty in imagining the scenarios, as evidenced by high reported vividness of the imagination for both the pleasant and neutral scenarios (7.7±1.2 vs 7.0±1.5, mean±SD, p = 0.08).

**Figure 3:**
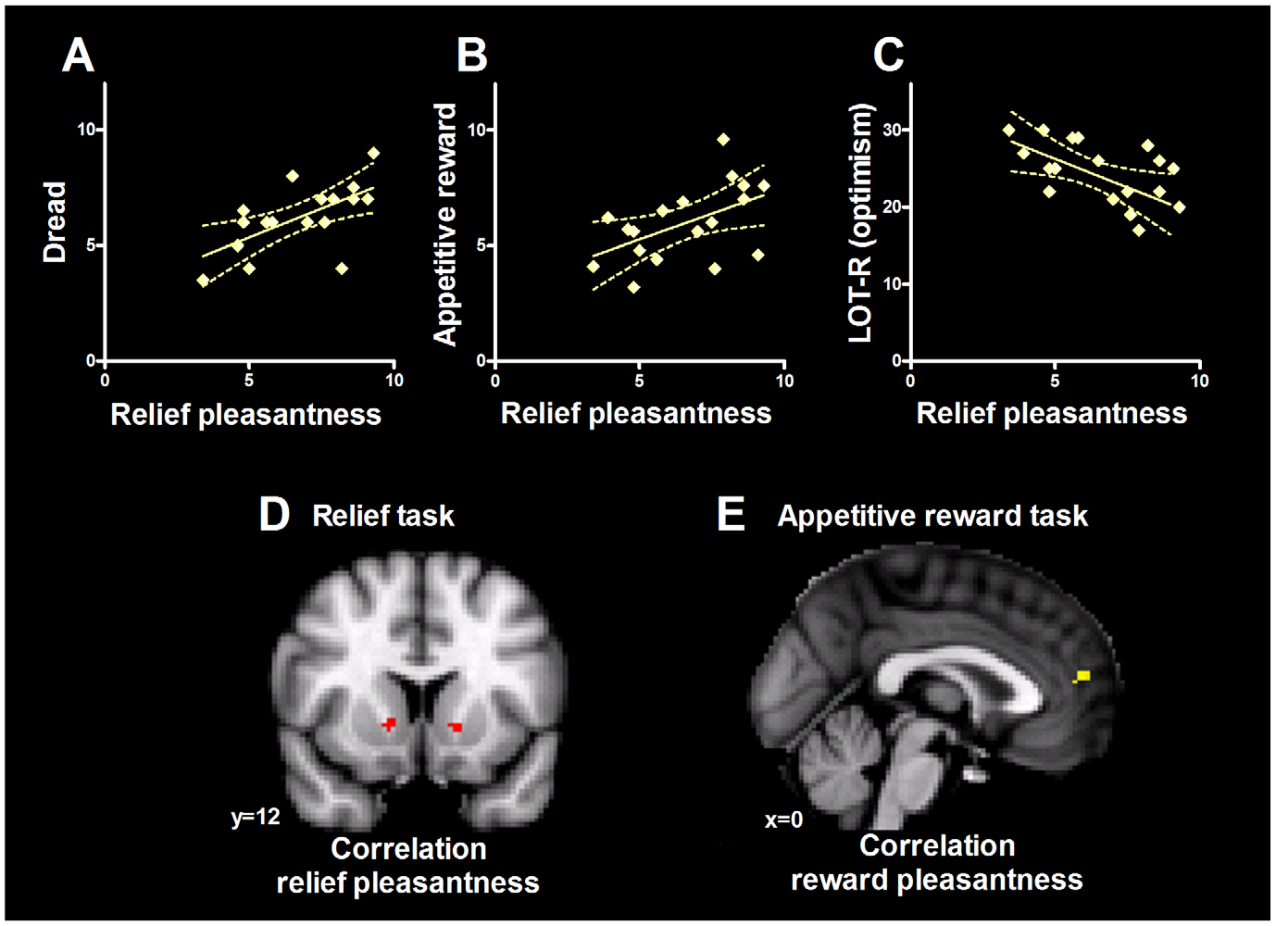
Correlation and regression analyses. Hedonic ratings of relief were significantly positively correlated with ratings of anticipatory dread in the relief task (A) as well as with hedonic ratings from the appetitive reward task (B). As hypothesised, relief hedonics also significantly covaried with optimism scores, such that more pessimistic participants reported higher relief pleasantness (C). Panel D shows a significant association between relief hedonics and accumbens (NAc) activation in the relief task. In contrast, hedonic ratings in the reward task did not covary with NAc activity; these ratings correlated with voxels in the rACC/mPFC region (E).

As illustrated in Figure 3A and B, reported pleasantness in the relief task was significantly correlated with pleasantness ratings in the appetitive reward task (r = 0.557, p = 0.02), and with debriefing reports of anticipatory dread elicited by the warning signal (r = 0.622, p = 0.008). As hypothesised, pessimism significantly correlated with both anticipatory dread (r = 0.512, p = 0.03) and with relief pleasantness (r = 0.568, p = 0.014; see Figure 3C), but not with pleasantness reports in the appetitive reward task (r = 0.223, p = 0.373). Similarly, reported dread did not significantly correlate with appetitive reward task hedonics (r = 0.358, p = 0.144). Since the intensity of the heat pain was tailored to each individual, there was little variance in the pain ratings, and as expected these ratings were not significantly correlated with any other measure.

### fMRI results

#### Main effect of relief and appetitive reward tasks

To determine the neural correlates specific to relief and appetitive reward in this study, we created the contrasts safety > control cue (‘relief’) and pleasant > neutral scenarios (‘appetitive reward’). Figure 2 illustrates the main findings (see also Table 2). The ‘relief’ contrast revealed significant peak activations in the ventromedial prefrontal cortex (vmPFC), rostral (pregenual) ACC, ventrolateral prefrontal cortex (vlPFC), bilateral anterior insula, and bilateral cerebellum. No significant main effect of relief was found in the ventral striatum. The ‘appetitive reward’ contrast yielded significant activations in regions previously associated with reward processing, notably the ventromedial prefrontal and orbitofrontal cortices, the ventral striatum, the posterior cingulate cortex, the left amygdala, and the bilateral hippocampal formation. Additional activations were found in the thalamus and brainstem. The activation in the vmPFC extended into the rostral and subgenual anterior cingulate cortex (ACC).

**Table 2:** Overview of peak MNI coordinates for areas of significant activation for the two main contrasts: pleasant > neutral scenarios (appetitive reward task) and safety > control cue (relief task).

Reward (pleasant > neutral scenarios)		x	y	z	Z
Ventromedial Prefrontal Cortex	R	2	56	2	5.04
	L	−2	50	0	4.65
Medial Orbitofrontal Cortex	R	4	42	−16	5.58
Rostral Anterior Cingulate	L	−4	44	4	5.38
	R	2	34	4	4.3
Subgenual Anterior Cingulate	L	−2	32	−14	4.56
Posterior Cingulate	L	−6	−56	12	4.59
	R	10	−54	2	3.43
Amygdala	L	−18	−6	−14	2.9
Parahippocampal Gyrus	L	−14	−36	−12	3.1
	R	34	−32	−14	3.08
Nucleus accumbens	L	−12	16	−6	3.2
Precuneous	R	10	−56	6	3.05
	L	−6	−56	10	4.67
Thalamus	L	−10	−6	6	3.02
	R	12	−18	2	3.03
Brainstem	L	−8	−34	−12	3.36
	R	4	−34	−12	3.29

Relief (safety > control cue)		x	y	z	Z
Rostral Anterior Cinguate	L	−4	42	4	3.58
	R	6	54	6	3.56
Ventromedial Prefrontal Cortex	R	2	58	14	3.36
	L	−2	58	10	3.17
Dorsomedial Prefrontal Cortex	R	4	44	44	3.43
	L	−2	44	46	3.03
Anterior Insula	R	32	18	−18	3.91
	L	−38	10	−14	3.79
Frontal Operculum	R	40	30	−6	3.35
Cerebellum	L	−36	−50	−40	3.7
	R	30	−52	−40	3.46

#### Common and differing activation for the relief and the appetitive reward tasks

Conjunction analysis of the relief and appetitive reward activation maps (thresholded as above using Z = 2.3, p<0.05) showed overlap between the two types of reward processing in the vmPFC and rACC. Significant differences between the two tasks were also identified; this analysis was restricted to areas showing a main effect of each task. Activity in the right anterior insula and bilateral cerebellum was higher during the relief task (safety cue>control cue) relative to the appetitive reward task (pleasant scenarios>neutral scenarios). The opposite contrast revealed significantly higher activity in the thalamus and posterior cingulate for appetitive reward relative to relief.

#### BOLD response covaries with hedonic responses

To further investigate the question of similarities and differences in the neural underpinnings between relief and appetitive reward, we tested for significant covariation between task-induced BOLD response and hedonic ratings of relief, dread and appetitive reward in two regions of interest: the vmPFC/rACC region identified as commonly activated by both tasks, and the *a priori* region of interest, the nucleus accumbens. These analyses revealed that only appetitive reward hedonics significantly correlated with task-induced BOLD response in voxels within the prefrontal overlap region (Figure 3F). No voxels within this region showed a significant relationship with relief hedonics during the relief task. Furthermore, the response (measured as maximum % change within the region of interest) during the appetitive reward task did not correlate significantly with the response during the relief task in the overlap region (r = 0.291, p = 0.241).

Similarly, only relief pleasantness and not appetitive reward pleasantness covaried significantly with activity within the left and right NAc (Figure 3E). This analysis confirmed the hypothesised correlation between positive hedonic feelings of relief and activation in the bilateral NAc. This finding extends previous reports of covariation between NAc activation and euphoria or expected pain relief [6], [31]. Our hypothesis specified that relief-related NAc activity would be related to the violation of negative expectation rather than with reward-related activity in general. The finding that voxels within the accumbens correlated with relief hedonics even when appetitive reward hedonics were included in the model is consistent with this hypothesis.

#### Brain activation covaries with trait pessimism

**Figure 4:**
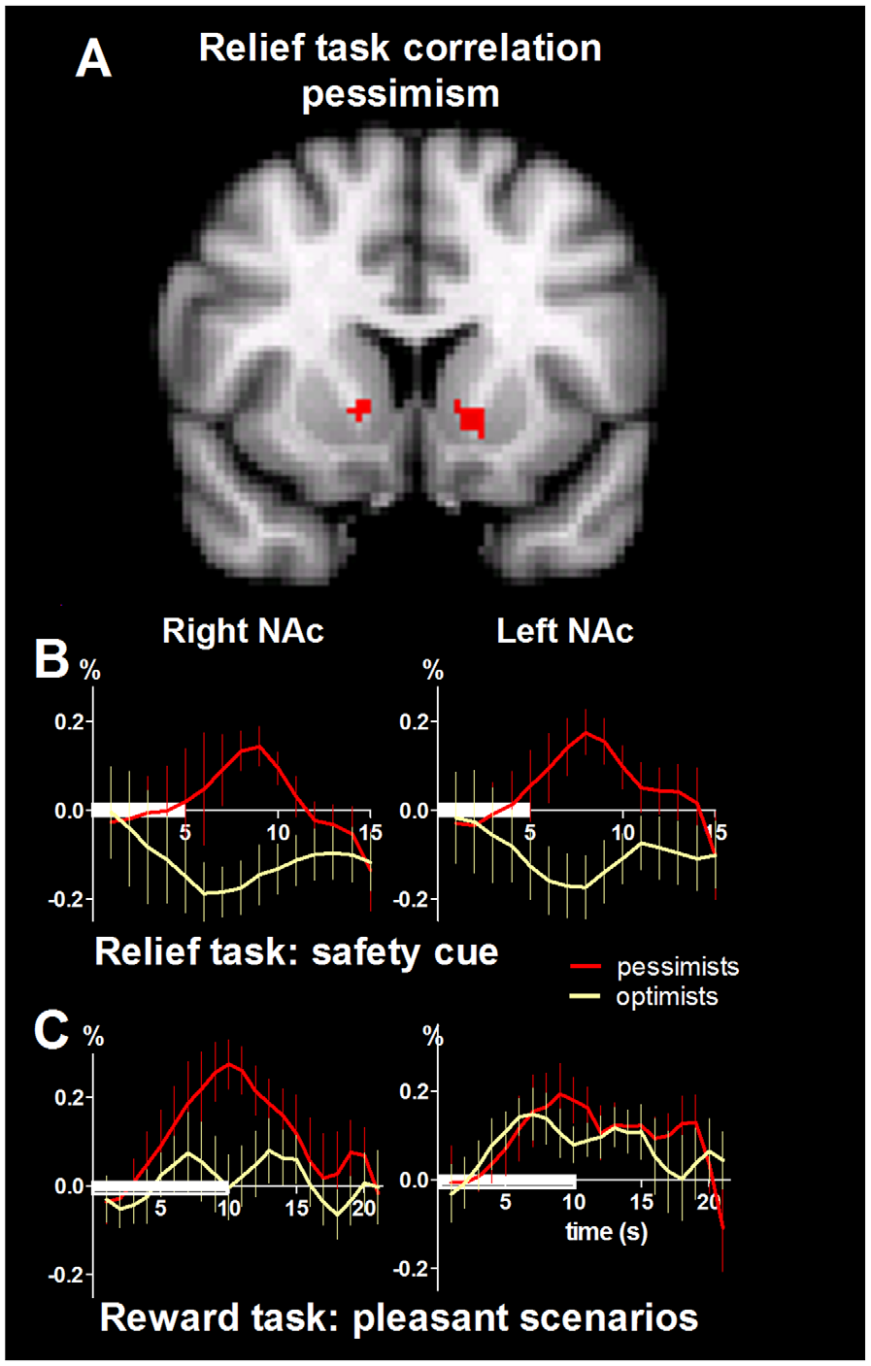
Pessimism and BOLD response to safety. A: Consistent with the observed effect of pessimism on relief hedonics, pessimism scores also correlated with voxels in the left and right NAc in the relief task. To illustrate this relationship, we split participants into more pessimistic and more optimistic groups using the median score on the LOT-R. The more pessimistic group (n = 7) showed higher relief pleasantness (p = 0.035, one-tailed), and the two groups showed opposite BOLD responses in the left and right nucleus accumbens (NAc) during unexpected safety (B). In contrast, both groups showed positive NAc responses during the pleasant scenarios in the appetitive reward task (C). Duration of stimuli are indicated by the white horizontal bars. Error bars indicate SEM. The MRI image shows the standard MNI brain.

As shown in the above regression analysis, the nucleus accumbens BOLD response during the relief task appeared to be more associated with the negative hedonic aspects of relief than with a purely appetitive reward response. Consistent with this, further regression analyses also found that voxels in the accumbens covaried with hedonic ratings of dread and with dispositional pessimism during relief. To illustrate this finding, we used a median split to divide participants into two groups: the more optimistic (n = 8) and the more pessimistic (n = 7), with pessimistic participants reporting significantly more pleasant relief (one-tailed t-test, p = 0.035). Mean peri-stimulus plots for the NAc were then generated for the groups; as depicted in Figure 4, opponent responses in the two subgroups of more and less pessimistic participants were found during the safety cue in the relief task, but not during the imagined pleasant scenarios in the appetitive reward task. The peri-stimulus plots generated for the control cue and neutral imagined scenarios (data not shown) also revealed similar responses in the two subgroups, consistent with a specific influence of disposition upon NAc responses to unexpected safety.

## Discussion

This study investigated hedonic and neural responses to safety from pain. We show that relief, an omission-induced reward, is related both to appetitive reward affect and to negative hedonics elicited by threat of pain (dread). Furthermore, hedonic feelings of relief and dread are stronger in participants with generalised negative expectations (pessimists). These influences on the relief response are underpinned by differing neural structures. Appetitive reward and relief task activation converged in the ventromedial prefrontal cortex, including the rostral anterior cingulate region. Voxels within this region also reflected individual differences in appetitive reward pleasantness. In contrast, and consistent with the concurrence of relief with positive violation of negative expectation, pleasantness elicited by safety from pain was related to the BOLD response in the nucleus accumbens. Importantly, the accumbens signal appeared to specifically reflect individual differences in responses to anticipation of the adverse event (dread, pessimism) but was uncorrelated to appetitive reward hedonics.

### BOLD response during relief and appetitive reward tasks

As expected, the appetitive reward task identified a main effect of task in a set of regions previously associated with primary and monetary rewards [31], [32], [33], [34], [35], [36], including the medial ventral and orbital prefrontal cortices, the ventral striatum, and the posterior cingulate cortex (Figure 2). These neuroimaging findings support the validity of the subjective ratings of increased pleasantness, rendering report bias unlikely. Therefore, simply imagining a positive emotional event is sufficient to generate brain activation patterns akin to those elicited by non-imagined events, as has been shown for negative emotional and other sensorimotor events [14], [15], [16].

Consistent with our prediction that appetitive and avoidance-induced rewards would activate partly the same neural networks, we identified common activation between the two reward tasks in the ventromedial prefrontal (vmPFC) and rostral anterior cingulate (rACC) cortices. This finding extends results from previous studies of monetary tasks where rewards and avoided losses similarly activate ventromedial prefrontal regions [8], [9]. Interestingly, the rostral ACC has been repeatedly implicated in the relief of pain by analgesic drugs, distraction and motor cortex stimulation [37], [38], [39]. This opioid-rich region [40] is also thought to drive placebo analgesia (expectation-induced relief) [41], [42], [43] and has been associated with reward prediction error [7].

### Pleasant and aversive influences on relief hedonics

As expected, individual differences in relief pleasantness were significantly associated with between-subject variability in the hedonic response in the appetitive reward task. This finding is consistent with a shared neural substrate influencing positive hedonics for relief as well as for appetitive rewards, identified here in the ventromedial prefrontal cortex. Voxels in this region also reflected individual differences in appetitive reward task pleasantness ratings. However, a similar relationship was not found for the relief task.

Individual differences in the relief response were also related to differences in responsiveness to the threat of pain, as measured by dread and pessimism scores. The considerable between-subject variability in the amount of dread experienced during anticipation of pain was explored by Berns and colleagues [44], who showed that some dread-prone participants find waiting for pain more aversive than pain itself. The significant correlation between dread and relief scores and dispositional pessimism reported here confirms our hypothesis that habitually expecting the worst enhances aversion of punishment cues, and consequently increases relief. This finding complements previous findings that pain ratings increase when participants' negative expectations are enhanced (and vice versa) [45], [46], [47].

BOLD response patterns in the relief and appetitive reward tasks also differed with regards to the nucleus accumbens. The ventral striatum, especially the accumbens (NAc), is known to encode salience and learning signals, and to show increased activation to uncertain rewards, especially when the current outcome is better than the expected outcome (reward prediction error) [7], [11]. Here, BOLD signal in the NAc correlated significantly with subjective reports of pleasantness during the relief task, but not during the appetitive reward task (Figure 3). This finding is consistent with the hypothesis that relief is influenced by two separable processes or neural systems; positive reward-induced hedonics, and negative hedonic feelings induced by expectation of pain. It appears that the relief-induced NAc signal reflects mainly affect related to negative expectations, since voxels within this region covaried with both dread and pessimism as well as relief, but were not associated with positive hedonic responses in the appetitive reward task. In fact, the NAc-relief pleasantness correlation remained significant even when appetitive reward hedonics were included in the regression model (GLM).

### Effect of disposition on hedonic experience and accumbens responses

Since surprise enhances emotions [48], we hypothesized that more negative expectations during the anticipation period would lead to greater relief and reward prediction errors in the NAc. As illustrated in Figure 4, only the relatively pessimistic participants in our cohort responded with an increase in NAc activity during the safety cue. This positive NAc response could reflect the increased salience of unexpected safety cues in pessimistic individuals. In contrast, and as predicted [11], [49], both the sense of relief and BOLD response in brain regions signalling prediction error were diminished in more optimistic participants. We believe the increased positive affect and safety cue-induced NAc signal exhibited by the more pessimistic participants in our study are a consequence of their surprise when the better (and less expected) outcome occurred. In contrast, more optimistic individuals, by virtue of their positive view of life, are predisposed to expect the better outcome. As the better outcome closely matches their expectation, both their sense of relief and the activity in brain regions signalling reward prediction error would be diminished [11], [13], [49], [50]. Individual NAc BOLD responses in a similar range (both negative and positive) were demonstrated in a previous study of monetary reward [51]. Note that the relationship identified here between outlook on life and hedonic and brain responses is based on group analyses of eighteen healthy volunteers who were not pre-screened to fall into the two extremes of the optimism-pessimism spectrum. Therefore, our findings do not allow conclusions on these extreme character traits, but avoid issues relating to sample bias.

Optimists are believed to derive more pleasure from life and to have a greater overall quality of life [52]. However, our results on relief represent a possible neural compensatory mechanism that enables a hedonic homeostasis [53] to be attained, such that pessimists' negative expectations can be ‘recompensed’ by more pleasant relief.

### Concluding remarks

In summary, we compared hedonic and BOLD responses during a relief task with responses during an appetitive reward task. Since relief is a type of reward, albeit non-appetitive, we expected and found covariation between responses in the two tasks. The pleasantness of relief correlated with appetitive reward task hedonics, and both tasks activated voxels within the ventromedial prefrontal cortex. Within this region of activation overlap we also identified voxels that reflected individual differences in appetitive reward task pleasantness. However, voxels within this region did not significantly reflect the pleasantness of relief. In contrast, relief pleasantness correlated with BOLD activity in the bilateral nucleus accumbens. We interpret these findings in light of the importance of positive prediction error for relief to occur: the more aversive the preceding event, the greater the subsequent relief. The accumbens-relief association reported here is consistent with a major role for negative expectations and pessimism in shaping relief hedonics.
